# Construction and verification of a nomogram model for predicting the risk of post-stroke spasticity: a retrospective study

**DOI:** 10.1080/07853890.2025.2604857

**Published:** 2025-12-23

**Authors:** Qian Xie, Jingling Zhu, Xuanling Cheng, Jianling Deng, Qing Song, Aiguo Xue, Shuxiong Luo

**Affiliations:** ^a^ Department of Tuina, Dongguan Hospital of Traditional Chinese Medicine, Guangzhou University of Chinese Medicine, Dongguan, China; ^b^ Department of Neurology, Dongguan Hospital of Traditional Chinese Medicine, Guangzhou University of Chinese Medicine, Dongguan, China; ^c^ Department of Acupuncture and Moxibustion, Dongguan Hospital of Traditional Chinese Medicine, Guangzhou University of Chinese Medicine, Dongguan, China

**Keywords:** Post-stroke spasticity, stroke, nomogram, predictors, LASSO

## Abstract

**Objective: **The aim of this study is to develop and validate a nomogram model for predicting the risk of post-stroke spasticity (PSS).

**Methods: **A retrospective study collected data from 366 stroke patients admitted to Guangzhou University of Chinese Medicine’s Dongguan Hospital between January 2022 and April 2025. PSS was defined as a Modified Ashworth Scale (MAS) score ≥1 within 3 months after stroke. The patients were divided into training and validation sets using a 7:3 ratio. Using multivariate logistic regression analysis and least absolute shrinkage and selection operator (Lasso) regression, a predictive model was built, and a nomogram was created for clinical application. The receiver operating characteristic (ROC) curve and calibration curve were plotted to assess the discrimination and calibration of the model. Decision curve analysis (DCA) and the clinical impact curve (CIC) were used to evaluate the model’s clinical applicability and usefulness.

**Results:**

LASSO-logistic regression analysis identified seven predictors associated with PSS: C-reactive protein, albumin, creatine kinase, fasting blood glucose, hyperlipidemia, sleep disorders, and manual muscle testing (MMT) score at admission. The model had an area under the curve (AUC) of 0.844 (95% CI: 0.793–0.896) in the training set and 0.842 (95% CI: 0.765–0.920) in the validation set, which means it was good at making predictions. The calibration curves showed excellent agreement between predicted and observed probabilities in the training set. Good calibration was maintained in the validation set, indicating only minimal overestimation of risk. DCA and CIC both agreed that the nomogram model could be used in a wide range of therapeutic situations.

**Conclusion:**

The nomogram based on routine clinical data in this study, after internal validation, can effectively predict the risk of PSS and provides a practical decision-making tool for clinicians. However, future multi-centre external validation is still required to confirm its broad applicability.

## 1. Introduction

Stroke ranks among the foremost causes of mortality and disability globally [1]. Statistics from the World Health Organisation indicate that it has emerged as the second biggest cause of world mortality [2]. Spasticity is an abnormal remodelling that occurs in the early recovery phase after stroke and can persist into the chronic phase. It is characterised by abnormal increases in muscle tone, manifesting as velocity-dependent hypertonic stretch reflexes and heightened tendon reflexes, ultimately resulting in postural deformities, such as common abnormal postures like elbow flexion contractures and lower limb extension with adduction [3,4]. According to epidemiological research [5], as many as 42.6% of people who have had a stroke will experience post-stroke spasticity (PSS), making it a common complication following stroke. PSS can seriously impede motor function and everyday life activities in stroke victims, causing substantial physical, psychological, and financial hardships for those impacted [6,7]. The onset of PSS exhibits variability, typically peaking between 1 to 3 months after a stroke. Studies indicate [8,9] that interventions such as Botulinum Toxin A (BoNT-A) should be initiated within 3 months of spasticity detection to prevent secondary complications. Similarly, it is noteworthy that, according to statistics [10], the medical costs for stroke survivors with spasticity are approximately four times higher than those for patients without spasticity. Early identification of PSS and timely, effective interventions can improve patient outcomes and reduce healthcare costs [11,12]. Therefore, the early prevention and treatment of PSS represent a critical focus in clinical research.

Prior research has indicated [13]that the site of haemorrhage or infarction within the basal ganglia, the extent of haemorrhage or infarction, and the NIHSS score are all independent risk factors for PSS. The occurrence of spasticity is the result of multifactorial interactions [14,15], which makes it challenging to predict its occurrence based on a single indicator. Currently, research on early predictive models for PSS remains limited, and the factors included are very few [13,16,17]. To address this gap, this study integrates patients’ clinical characteristics, underlying diseases, laboratory indicators, and other clinical baseline data for the early prediction of PSS risk. We aim to construct a comprehensive, easy-to-use, visual predictive model for PSS that will help physicians identify high-risk patients early on. Additionally, this model will provide a standardised predictive tool for the field of stroke rehabilitation medicine, which will encourage additional research to be conducted in this particular area.

## 2. Methods

### 2.1. Study designs and participants

This study was conducted in accordance with the principles indicated in the Declaration of Helsinki, and it was granted ethical permission by the Ethics Committee of Dongguan Hospital, which is affiliated with Guangzhou University of Chinese Medicine (Approval number: PJ[2025] No. 91). The Ethics Committee exempted our study from obtaining informed consent from participants because our research was conducted as a retrospective analysis based on relevant data from an existing database.

A retrospective collection of data was performed on 366 patients who were hospitalised in the Rehabilitation Department following stroke at Dongguan Hospital of Guangzhou University of Chinese Medicine from January 2022 to April 2025. Each subject underwent a spasticity assessment of the upper limbs, lower limbs, and trunk using the Modified Ashworth Scale (MAS) within 3 months after the onset of acute stroke. any patient who scored ≥1 in any joint on the MAS was considered to have spasticity. Based on these scores, stroke patients were categorised into the PSS group and the Non-PSS group. Inclusion Criteria: Meeting the diagnostic criteria for cerebrovascular disease established at the Fourth National Conference [18], confirmed by head CT or MRI; Age ≥ 18 years. Exclusion criteria: Patients with transient ischemic attacks; Patients with severe language impairment; Patients with consciousness disturbances; Having other serious health problems at the same time, such as cancerous tumours, severe liver or kidney issues, major injuries, breathing problems, or severe heart disease; History of severe psychiatric symptoms; Missing data.

### 2.2. Clinical data acquisition

Through literature review and clinical expertise, 26 easily accessible clinical variables were selected. Clinical data were systematically extracted from the patients’ electronic medical records, including: (1) Demographics: Age, gender, medical history (hypertension, diabetes, hyperlipidemia),smoking history. (2) Clinical features: type of stroke (hemorrhagic, infarction), location of occlusion (cerebral cortex, cerebellum, thalamus, basal ganglia, brainstem), side of infarction or hemorrhage (left side, right side), large-area cerebral infarction. (3) Laboratory Indicators: White blood cells (WBC), C-reactive protein (CRP), albumin (ALB), fasting blood glucose (FBG), glycosylated hemoglobin (HbA1c), creatine kinase (CK), lactate dehydrogenase (LDH), potassium, magnesium, and calcium. (4) Sleep disorders were assessed using the Pittsburgh Sleep Quality Index (PSQI). This scale consists of 18 items grouped into 7 components, with a total score of 21. A PSQI score greater than 7 at admission indicated a sleep disorder, while a score of 7 or below indicated good sleep quality. (5) Depression was assessed using the Self-Rating Depression Scale (SDS) [19]. The scale contains 20 items rated on a 4-point Likert scale (1–4 points), with 10 items being reverse-scored. Patients with an SDS score of 53 or above at admission were identified as exhibiting depressive symptoms. (6) Muscle strength was routinely quantified using the Manual Muscle Testing (MMT) 0–5 point scoring system [20]. Within this system, a score of 0–3 indicates moderate to severe muscle strength deficit, while a score of 4–5 indicates good to normal muscle strength. (7) The activities of daily living (ADL) of the patients were evaluated using the Barthel Index. Based on scores, it is divided into three levels: Independent (100 points), Basic Self-Care (61 to 99 points), and Poor Self-Care Ability (less than 60 points). It comprises 10 different aspects. Higher scores indicate less dependence and greater independence. (8) The NIHSS has 15 elements, yielding a total score between 0 and 42. Elevated scores signify greater neurological impairment severity. The scoring categorization is delineated as follows: A score below 8 signifies mild impairment, whereas a score of 8 or more denotes significant impairment. (9) The Nutritional Risk Screening 2002 (NRS-2002) is utilized to evaluate nutritional risk in adult hospitalized patients. A number of less than 3 signifies no nutritional risk, a score of 3 or greater denotes low nutritional risk, and a score of 5 or greater indicates significant nutritional risk [21].

We excluded patients with >10% missing data in candidate predictors. Remaining missing values (<10%) were imputed *via* multiple imputation in SPSS. Sensitivity analyses verified that the findings were robust to both missing data and the imputation method (Supplementary Material 1).

### 2.3. Statistical analysis

The R software (version 4.4.3) and the SPSS software (version 27.0) were the primary tools utilised in the implementation of statistical studies. 366 participants were each subjected to descriptive statistical analysis.

(1) For continuous measurements that followed a normal distribution, the mean ± standard deviation (SD) was the method that was utilised to report the data. On the other hand, categorical data were presented as n (%). The median and interquartile range were used to represent data that were not normally distributed. Chi-square tests were utilised for the evaluation of categorical variables, whereas the Mann-Whitney U test was utilised for the evaluation of data distributions that were not normally distributed. (2) To ensure the fairness and comparability of the Least Absolute Shrinkage and Selection Operator (LASSO) regression, all continuous predictor variables were standardised using the Z-score method *via* the scale function in R (mean = 0, standard deviation = 1) prior to model fitting. To evaluate the model’s stability and generalizability, ten-fold cross-validation was employed to determine the optimal parameters, and internal validation with bias correction was performed using 1,000 Bootstrap resamples. (3) Multicollinearity diagnostics, calculating the Variance Inflation Factor (VIF), were performed on all candidate predictor variables. Subsequently, LASSO regression was employed for feature selection. For the purpose of determining the ideal punishment coefficient, the lambda value related to the cross-validation error that was within one standard deviation of the minimum error was utilised. Finally, the variables selected by LASSO were incorporated into a multivariable logistic regression model, and multicollinearity was re-examined to ensure the stability of the parameter estimates. (4) The discrimination ability of the model was evaluated using the Receiver Operating Characteristic (ROC) curve and the Area Under the Curve (AUC). The goodness-of-fit and calibration of the model were comprehensively assessed using the Hosmer-Lemeshow test, calibration curves, and the Brier score. Furthermore, Decision Curve Analysis (DCA) and the Clinical Impact Curve (CIC) were employed to evaluate the clinical net benefit of the model across different decision thresholds, thereby exploring its clinical utility value.

## 3. Results

### 3.1. Patient characteristics

**Figure 1.**
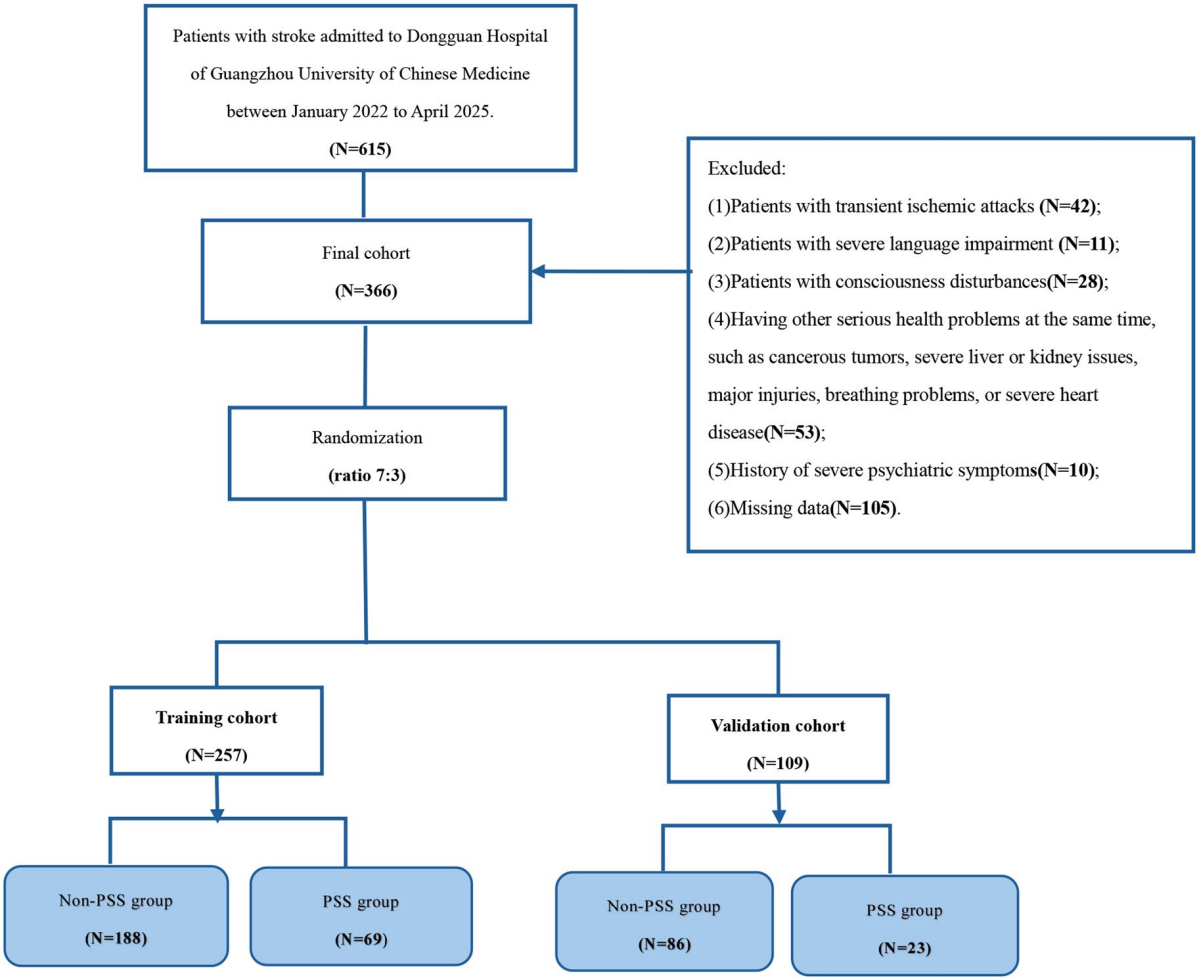
**Flowchart**.

Of the 615 stroke patients included in this study, 249 were excluded. In the end, 92 cases (25.86%) of the 366 participants in the study were diagnosed with PSS. Following a 7:3 random sampling ratio, 257 instances were allocated to the training set and 109 cases to the validation set. Figure 1 displays a flowchart. Furthermore, Table 1 outlines the traits of the PSS and Non-PSS groups.

**Table 1.** Baseline characteristics of the Non-PSS and PSS groups.

*Variables*		Total (*n* = 366)	Non-PSS (*n* = 274)	PSS (*n* = 92)	*p* value
**Demographics**					
Age, years, mean ± SD		61.69 ± 11.53	62.04 ± 11.77	60.65 ± 10.79	0.317
Gender, n (%)	Male	267(73.0%)	199(72.6%)	68(73.9%)	0.810
	Female	99(27.0%)	75(27.4%)	24(26.1%)	
**Medical history**					
Hypertension, n(%)		305(83.3%)	229(83.9%)	76(82.6%)	0.829
Diabetes, n(%)		126(34.4%)	96(35.0%)	30(32.6%)	0.672
Hyperlipidemias, n(%)		70(19.1%)	60(21.9%)	10(10.9%)	**0.020**
Smoking, n(%)		106(29%)	81(29.6%)	25(27.2%)	0.662
**Clinical features**					
Types of stroke, (n, %)	Infarction	274(74.9%)	207(75.5%)	67(72.8%)	0.603
	Hemorrhage	92(25.1%)	25(27.2%)	67(24.5%)	
Location of occlusion, (n, %)	Cerebral cortex	121(33.1%)	100(36.5%)	21(22.8%)	**0.020**
	Cerebellum	14(3.8%)	13(4.7%)	1(1.1%)	
	Thalamus	31(8.5%)	23(8.4%)	8(8.70%)	
	Basal ganglia	157(42.9%)	105(38.3%)	52(56.5%)	
	Brainstem	43(11.7%)	33(12%)	10(10.9%)	
Side of infarction or hemorrhage, (n, %)	Left	178(48.6%)	130(47.4%)	48(52.2%)	0.432
	Right	188(51.4%)	144(52.6%)	44(47.8%)	
Large area of infarction, (n, %)	Yes	108(29.5%)	77(28.1%)	31(33.7%)	0.309
	No	258(70.3%)	197(71.9%)	61(66.3%)	
MMT, (n, %)	0–3 level	153(41.8%)	130(47.4%)	23(25.0%)	**<0.001**
	4–5 level	213(58.2%)	144(52.6%)	69(75%)	
Sleep disorders, (n, %)		90(24.6%)	45(16.4%)	45(48.9%)	**<0.001**
Depression, (n, %)		38(10.4%)	20(7.30%)	18(19.6%)	**<0.001**
ADL, (n, %)	100	34(9.3%)	23(8.4%)	11(12.0%)	0.740
	99–61	215(58.7%)	157(57.3%)	58(63.0%)	
	<60	117(32.0%)	23(25.0%)	94(34.3%)	
NRS, (n, %)	<3	186(50.8%)	135(49.3%)	51(55.4%)	0.227
	≥3	136(37.2%)	103(37.6%)	33(35.9%)	
	≥5	44(12.0%)	36(13.1%)	8(8.7%)	
NIHSS, (n, %)	<8	200(54.6%)	135(49.3%)	65(70.7%)	**<0.001**
	≥8	166(45.4%)	139(50.70%)	27(29.3%)	
**Inspection results**					
White blood cell, mean ± SD		6.91 ± 1.92	6.89 ± 1.93	6.97 ± 1.92	0.731
C-reactive protein, Median ± SD		4.50 ± 4.12	3.73 ± 3.89	6.79 ± 3.95	**<0.001**
Albumin, mean ± SD		40.30 ± 4.02	39.96 ± 3.12	41.30 ± 5.85	**0.038**
HbA1c, mean ± SD		6.11 ± 1.65	6.14 ± 1.42	6.04 ± 2.22	0.678
Fasting blood glucose, mean ± SD		5.78 ± 1.55	5.17 ± 1.35	5.99 ± 2.03	0.226
Creatine kinase, mean ± SD		89.46 ± 61.50	80.05 ± 51.87	111.48 ± 77.71	**<0.001**
Lactate dehydrogenase, mean ± SD		180.18 ± 42.60	180.41 ± 44.29	179.48 ± 37.35	0.857
Potassium, mean ± SD		3.91 ± 0.41	3.90 ± 0.42	3.94 ± 0.38	0.417
Magnesium, mean ± SD		0.86 ± 0.13	0.86 ± 0.12	0.87 ± 0.17	0.591
Calcium, mean ± SD		2.33 ± 0.13	2.33 ± 0.12	2.35 ± 0.13	0.190

**Note:** Bold indicates *p* < 0.05.

**Abbreviations:** MMT, Manual Muscle Testing; ADL, Activities of Daily Living; NRS, Nutritional Risk Screening; NIHSS, National Institute of Health Stroke Scale; HbA1c, Glycated haemoglobin; SD, Standard deviation.

### 3.2. Selection of predictive factors for PSS

**Figure 2.**
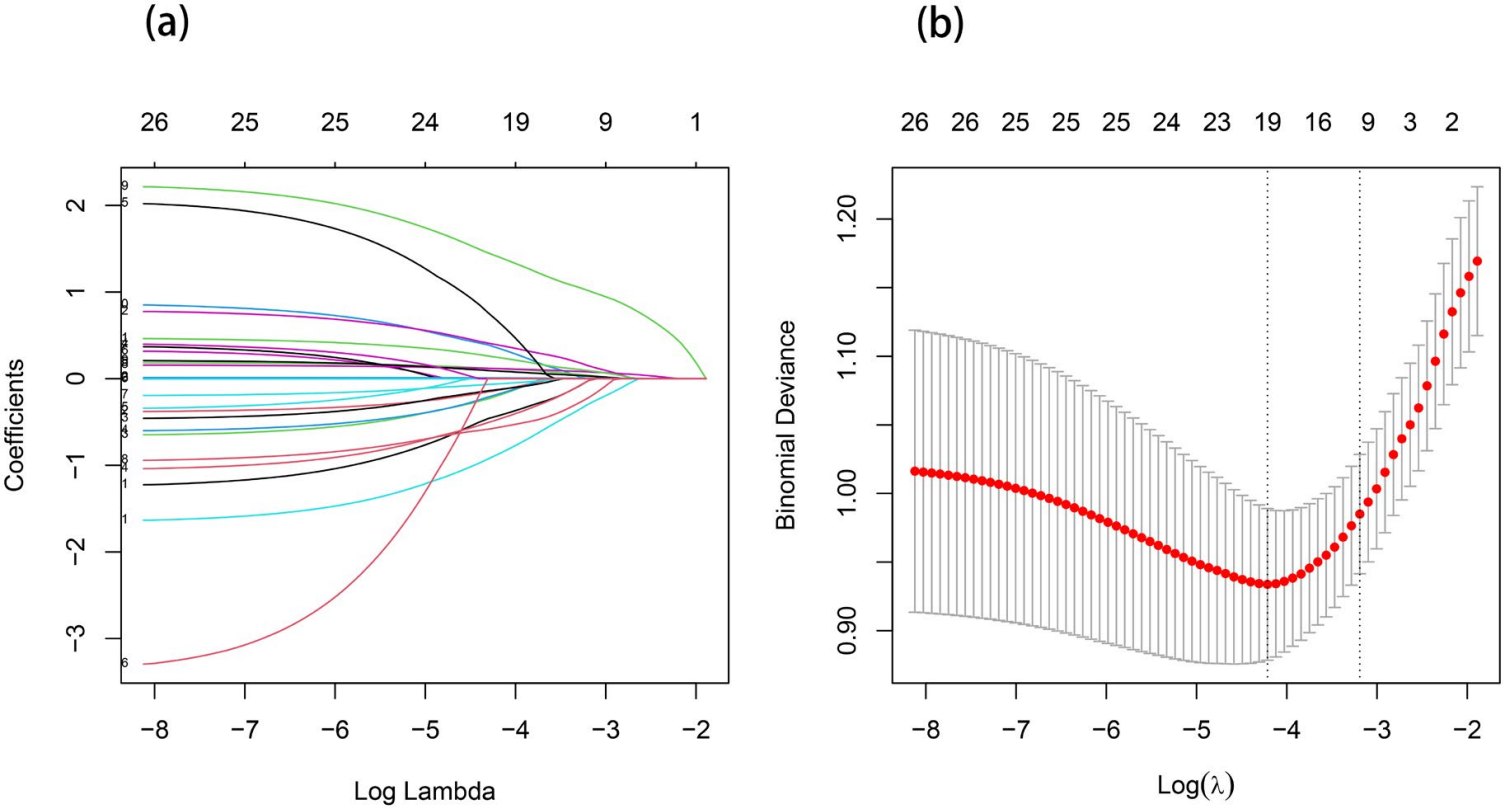
LASSO Regression. (a) Lasso regression coefficient distribution curve. (b) The optimal parameter (lambda) selection in the model employed ten-fold cross-validation using a minimisation criterion. Vertical dashed lines are drawn to respectively indicate lambda.min and lambda.1se (the one standard error criterion) to determine the optimal predictive variables.

Multicollinearity was assessed for all variables (Supplementary Material 2, Supplementary Table 1). LASSO regression was used to select predictive factors, employing ten-fold cross-validation and a minimization criterion to determine the optimal coefficient λ, which led to the selection of nine non-zero coefficient predictive variables, including hyperlipidemia, sleep disorders, MMT score, NIHSS score, location of occlusion, CRP, ALB, FBG, and CK (Figure 2a and 2b). After that, a multivariable logistic regression analysis was carried out on the predictive factors that were chosen by the use of LASSO regression. The results suggested that CRP (OR: 1.159, 95% CI: 1.070 ∼ 1.256), ALB (OR: 1.135, 95% CI: 1.037 ∼ 1.242), CK (OR: 1.009, 95% CI: 1.004 ∼ 1.015), FBG (OR: 1.370, 95% CI: 1.118 ∼ 1.680), hyperlipidemia (OR: 0.279, 95% CI:0.096 ∼ 0.813), along with sleep disorders (OR: 4.819, 95% CI:2.343 ∼ 9.912) and MMT score (OR: 0.284, 95% CI:0.130 ∼ 0.618), were recognised as independent factors impacting PSS (Table 2). The Variance Inflation Factor (VIF) values for all predictors were significantly lower than the threshold of 5, suggesting the absence of severe multicollinearity (Supplementary Material 2, Supplementary Table 2).

**Table 2.** Multivariable logistic regression results for predicting PSS patients.

Variables	OR	95%CI	*p*
CRP	1.159	1.070 ∼ 1.256	<0.001
ALB	1.135	1.037 ∼ 1.242	0.006
CK	1.009	1.004 ∼ 1.015	<0.001
FBG	1.370	1.118 ∼ 1.680	0.002
Hyperlipidemia	0.279	0.096 ∼ 0.813	0.019
Sleep disorder	4.819	2.343 ∼ 9.912	<0.001
MMT score			
Good to normal muscle strength (4–5 score)	0.284	0.130 ∼ 0.618	0.002

OR: Odds Ratio, Cl: Confidence Interval.

### 3.3. Construction of the nomogram

**Figure 3.**
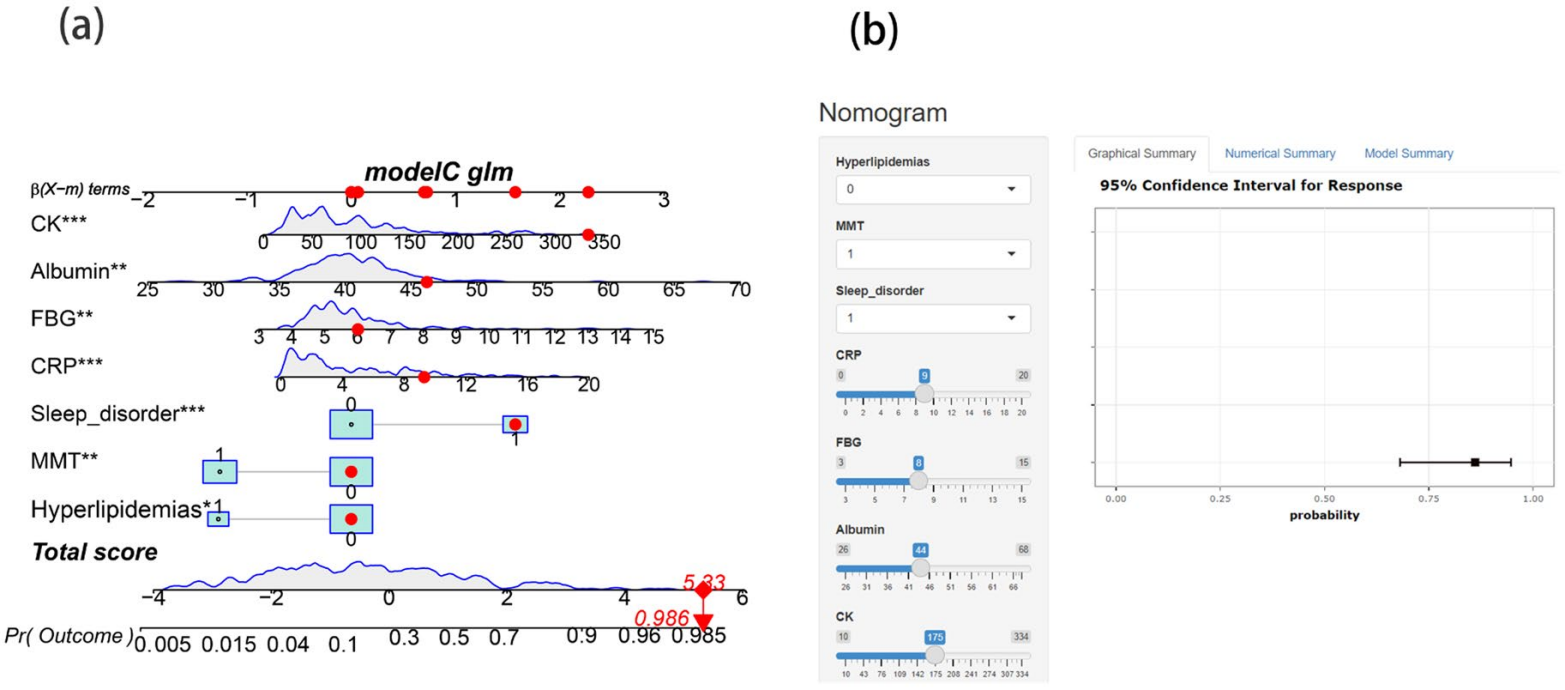
Nomogram prediction model for patients with PSS.

Using the findings of multivariable logistic regression, a nomogram for PSS patients was created (Figure 3a). Each variable value is assigned a corresponding score. After summing the scores of all variables to obtain a total score, a vertical line is drawn from this total score to estimate the probability of PSS occurrence. To ensure transparency and allow for offline verification, the complete model formula is provided in Supplementary Material 3. The formula includes all predictor coefficients (β values) and the intercept term, enabling independent validation and external application of our model. To illustrate the clinical applicability of the scoring system, a representative real-patient case was randomly selected from our study cohort. This patient had a CK level of 340 U/L, albumin level of 46 g/L, FBG of 6.0 mmol/L, CRP level of 9 mg/L, presence of sleep disorders, a MMT grade of 0–3, and hyperlipidemia. According to our prediction model, this patient’s estimated risk of developing PSS was high, approximately 92.5%. To facilitate clinical use by physicians and patients, we have developed a web-based version of the nomogram (Figure 3b), which is publicly available at: https://newpredict.shinyapps.io/DynNomapp2/.

**Figure 4.**
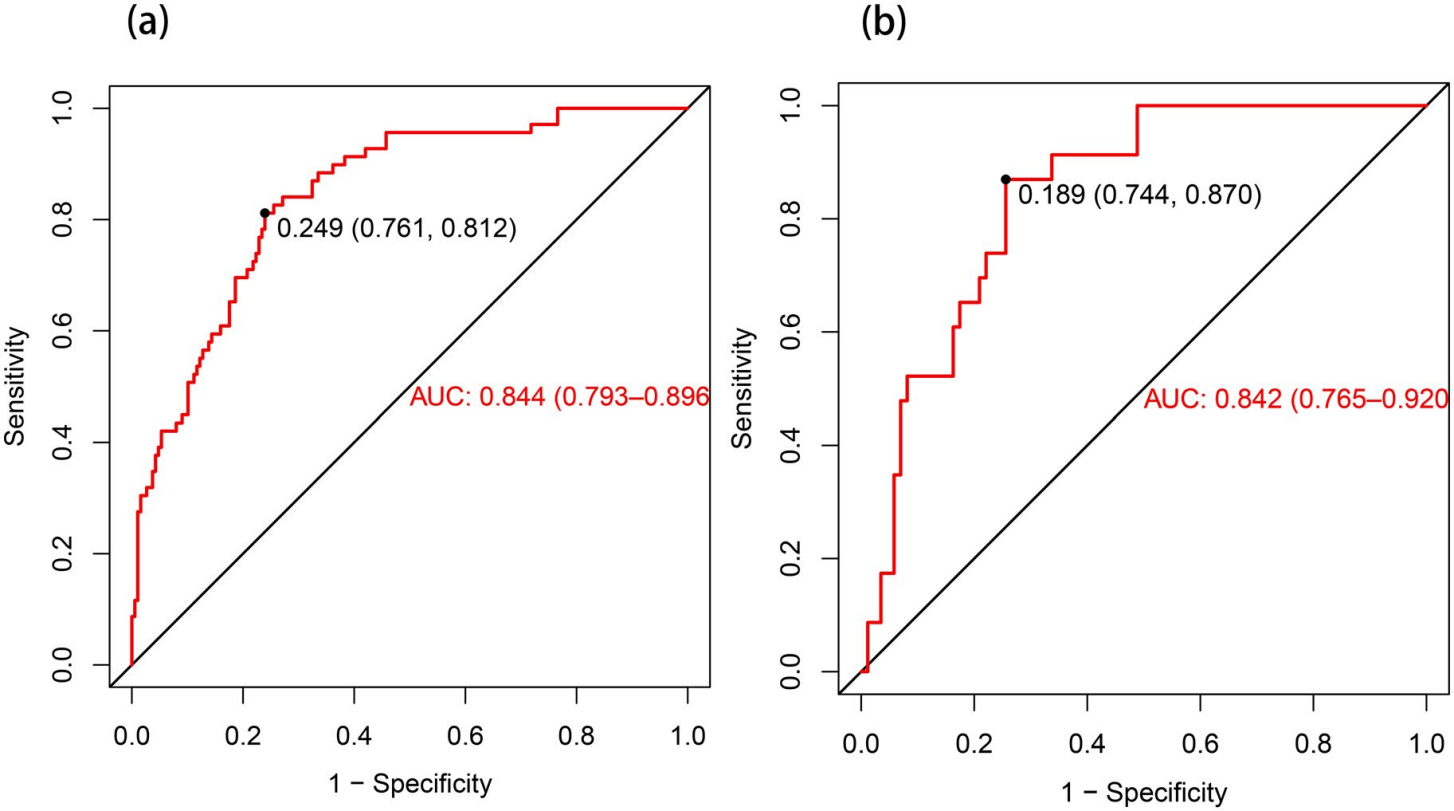
ROC curves for the model in both the training (a) and validation (b) sets.

The AUC for the training set and validation set was 0.844 (95% CI 0.793–0.896) and 0.842 (95% CI 0.765–0.920), respectively. These values exceed 0.7, suggesting that the model exhibits high predictive accuracy and good discrimination ability (Figure 4a and 4b). The optimal discrimination threshold was determined by maximising the Youden’s index. In the training set, a Youden’s index of 0.573 corresponded to an optimal cutoff value of 0.249, yielding a sensitivity of 81.2% and a specificity of 76.1%. In the validation set, a Youden’s index of 0.614 corresponded to an optimal cutoff value of 0.189, resulting in a sensitivity of 87.0% and a specificity of 74.4%. These consistent results suggest that the nomogram model exhibits stable discriminative performance and holds potential for clinical application. The high negative predictive values (91.7% and 95.5%) are particularly noteworthy, suggesting the model’s strong capability to reliably rule out the condition. The comprehensive performance metrics are summarised in Table 3.

**Table 3.** Diagnostic performance indicators of the model based on preset clinical Decision thresholds.

Cohort	Cut-off Value	Sensitivity	Specificity	PPV	NPV	AUC (95% CI)
**Training set**	0.249	81.2%	76.1%	55.4%	91.7%	0.844 (0.793–0.896)
**Validation set**	0.189	87.0%	74.4%	51.6%	95.5%	0.842 (0.765–0.920)

CI, confidence interval; PPV, positive predictive value; NPV, negative predictive value.

To address concerns regarding the potential inclusion of transient acute hypertonia and to rigorously evaluate the robustness of our nomogram, we performed a sensitivity analysis by applying stricter diagnostic criteria for PSS (Supplementary Material 4). Specifically, PSS was redefined using a MAS score of ≥1+ at 3 months post-stroke. The nomogram’s predictive performance remained high under this stricter definition, with an AUC of 0.861 (95% CI: 0.821–0.901), which is comparable to the AUC of 0.855 (95% CI: 0.814–0.895) obtained from the original model using the standard MAS ≥1 threshold. The minimal difference in AUC values indicates that the predictive accuracy of our nomogram was not significantly affected by the stringency of the PSS diagnostic threshold, demonstrating the model’s robustness.

### 3.4. Internal validation strategy

To evaluate the generalization ability and robustness of the predictive model, this study employed two internal validation methods (Supplementary Material 5): 10-fold cross-validation and bootstrap resampling. The mean AUC obtained from the 10-fold cross-validation was 0.840. After 1,000 bootstrap resamples for optimism correction, the model achieved a C-index of 0.823. These results indicate a reliable performance estimate for the model.

### 3.5. Validation of the nomogram

**Figure 5.**
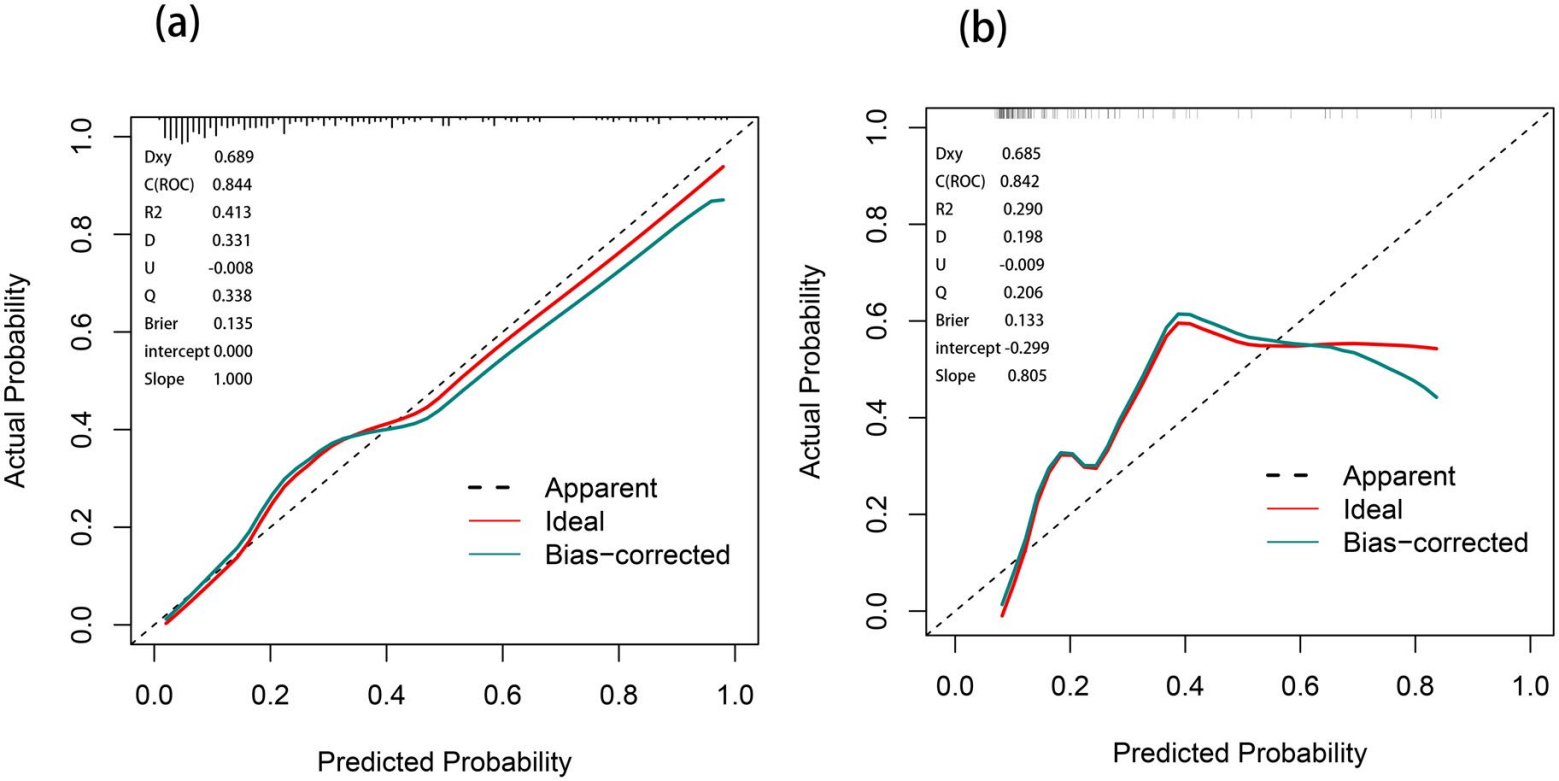
Calibration curves for the PSS nomogram prediction.

The Hosmer-Lemeshow test was used to evaluate the goodness-of-fit of the model. The results from both the training set (*p* = 0.944) and the validation set (*p* = 0.157) indicated no significant difference between the model’s predicted probabilities and the observed actual outcomes. The calibration curves presented in Figure 5a and 5b demonstrate the model’s excellent calibration performance. In the training set, with a calibration slope of 1.000, an intercept of 0.000, and a Brier score of 0.135, the predicted probabilities show an almost perfect alignment with the actual outcomes. In the validation set, the calibration slope is 0.805, the intercept is −0.299, and the Brier score is 0.133. These values indicate a slight systematic overestimation of risk by the model, a trend that becomes more pronounced within the high-risk range.

**Figure 6.**
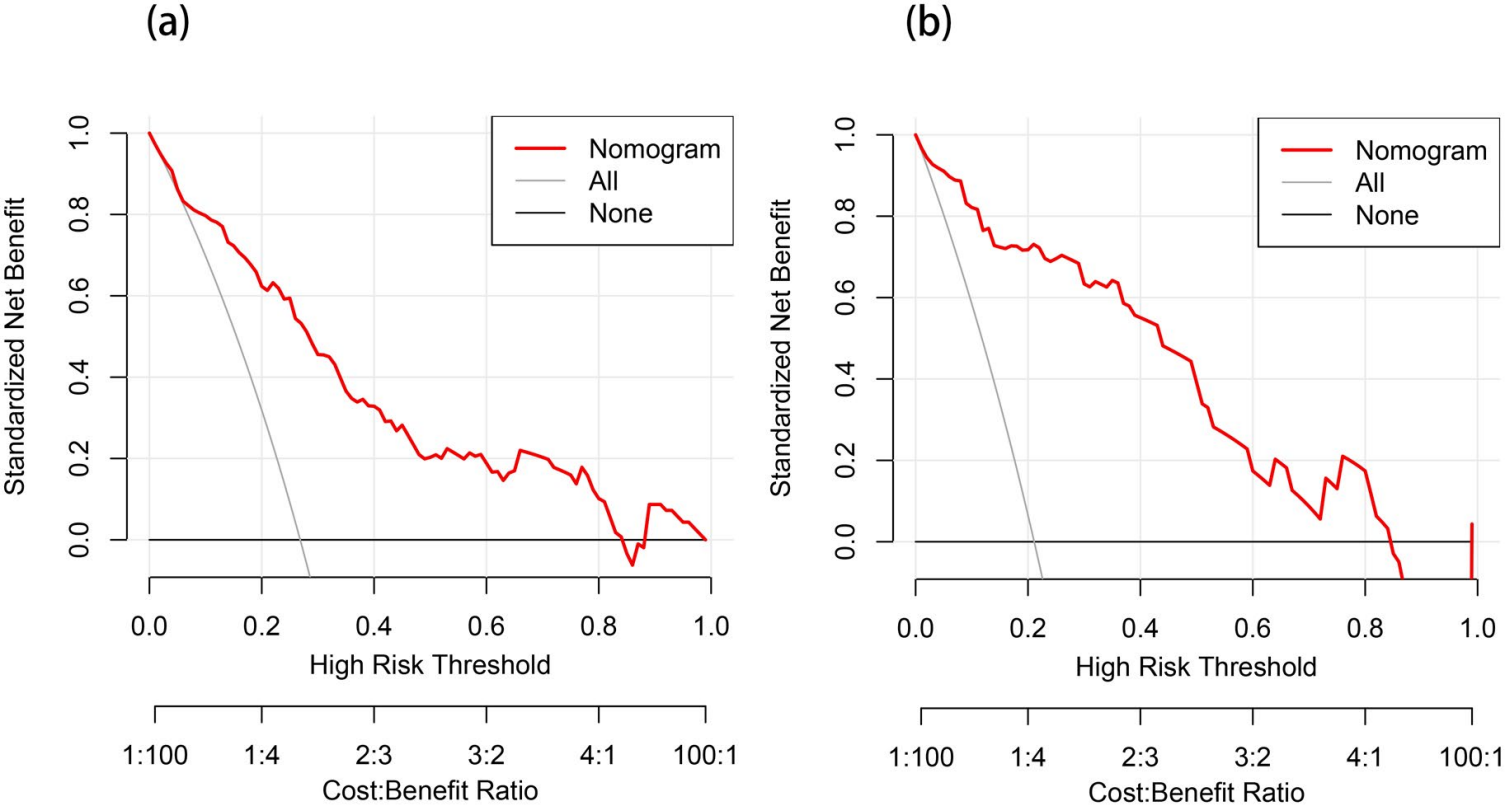
DCA for the PSS nomogram prediction.

We further evaluated the clinical utility of the nomogram using DCA and CIC. As shown in Figure 6, in the training set (a), when the threshold probability ranged from approximately 10% to 75%, and in the validation set (b), when the threshold probability ranged from approximately 5% to 70%, the standardized net benefit obtained by using the nomogram was higher than the strategies of “treat all” or “treat none”, demonstrating its broad clinical applicability.

**Figure 7.**
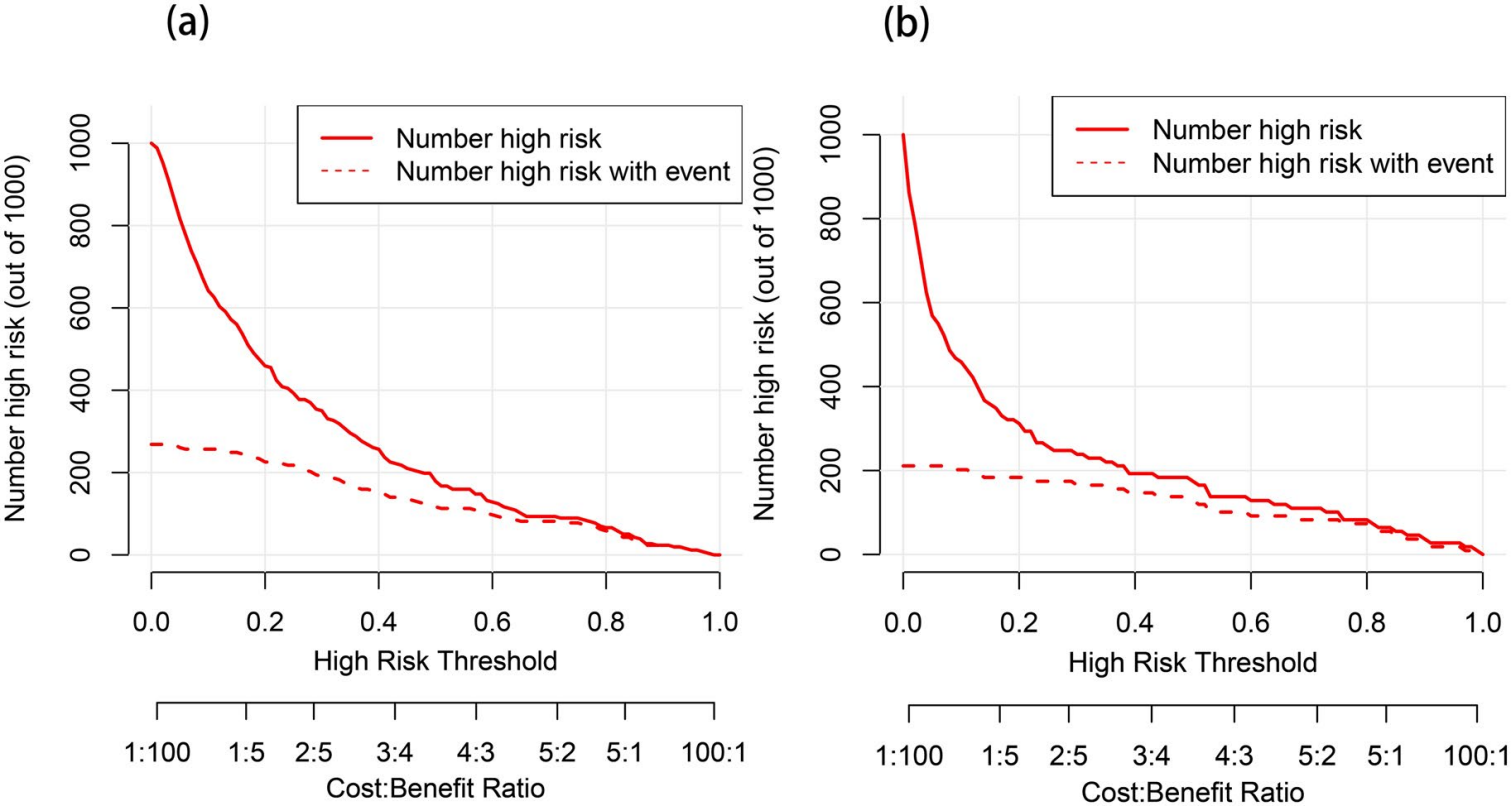
CIC for the PSS nomogram prediction.

The CIC (Figures 7a and 7b) revealed the excellent calibration characteristics of the model. When the high-risk threshold was set at approximately 65% or higher, the curve of the number of high-risk patients predicted by the model showed a trend of high proximity to the curve of the number of actual high-risk patients who experienced the event. This indicates that within this threshold interval, the model exhibits superior calibration, controlling the over-prediction (false positive) phenomenon at a very low level. Consequently, if clinicians choose this threshold to define the high-risk population, they can efficiently and accurately identify the true target patients with PSS who require intervention while maintaining a low Number Needed to Treat (NNT), thereby confirming the model’s high reliability and feasibility in practical applications.

## 4. Discussion

Currently, the most common treatments for PSS in clinical practice include pharmacological interventions [22], intramuscular injection of botulinum toxin [23], physical therapy [24], and surgery [25]. However, there is a significant gap between current clinical treatment methods and patients’ expectations for meaningful recovery [26], creating an urgent need for new strategies to prevent spastic hemiplegia following stroke. In recent years, the nomogram has been extensively employed as a graphical statistical instrument to forecast the occurrence of diseases by applying key parameters [27]. By making complex mathematical models more accessible, physicians can facilitate rapid and intuitive prediction and judgment processes. Our study developed and internally validated a nomogram-based predictive model for the purpose of determining the risk of spasticity in post-stroke patients. The results identify CRP, ALB, CK, FBG, hyperlipidemia, sleep disorders, and MMT score as key predictors for PSS. Moreover, the model demonstrates good predictive capability, excellent calibration performance, and high clinical utility.

In stroke patients, elevated levels of CRP are considered one of the important biomarkers associated with disease severity, prognosis, and the risk of complications [28–30]. Studies have found that increased apoptosis of cells in brain tissue and elevated levels of inflammatory factors are major pathological factors exacerbating spasticity after stroke [31]. Given that systemic inflammation following stroke may represent a potential therapeutic target, it is of the utmost importance to gain a deeper understanding of the connection between peripheral inflammation and PSS in a clinical setting. Our multivariate logistic regression analysis identified a higher serum albumin level (OR = 1.135) as an independent factor associated with an increased risk of PSS. Previous studies have generally indicated that low serum albumin levels are associated with poor prognosis in patients with stroke. However, a recent large-scale prospective study involving 5,111 stroke patients [32] revealed a unique U-shaped relationship between serum albumin and neurological functional outcomes. This finding suggests that albumin levels are not simply “the higher, the better.” Indeed, when levels exceed a specific threshold (approximately >42.2 g/L), further increases may be associated with an elevated risk of adverse outcomes. The relatively high albumin levels observed in our study might lie on the ascending branch of this U-shaped curve. This finding appears to contradict the traditional perspective and implies that the potential relationship between elevated serum albumin and the occurrence of PSS may be more complex than previously understood.

This study identified an association between CK levels and the risk of PSS. CK serves as a non-specific marker of muscle damage or stress. Recent studies [33,34]have found that serum CK is elevated in stroke patients, which may reflect abnormal muscle activity, immobilisation, or subclinical muscle injury during the acute phase and recovery process after stroke. These pathological processes might exacerbate the occurrence and development of spasticity by triggering inflammatory responses and metabolic disturbances. However, there is currently a lack of longitudinal studies directly investigating CK as a prospective predictor for PSS. The exact pathophysiological mechanisms and their potential as a reliable biomarker still require further investigation and clarification in future research.

Prior research has demonstrated [35] that diabetes is a risk factor for upper limb spasticity in hemiplegic patients following a stroke, which is consistent with our findings. Our research indicates that elevated FBG is an independent risk factor for PSS. This may occur through mechanisms such as oxidative stress, inflammatory responses, and disruption of the blood-brain barrier, which exacerbate brain injury and lead to neuronal apoptosis [36]. These factors can affect neuromuscular conduction and further impair the function of central inhibitory pathways, such as the corticospinal tract, resulting in an imbalance in the regulation of spinal stretch reflexes, which intensifies increased muscle tone and abnormal motor patterns [37], thereby raising the risk of spasticity.

This study unexpectedly identified hyperlipidemia as a protective factor against PSS. This seemingly paradoxical finding may reveal the complex role of lipid metabolism in neural recovery. The underlying mechanisms could be associated with the neuroprotective effects of statins [38] and the beneficial functions of specific lipid components, such as high-density lipoprotein cholesterol (HDL-C). Furthermore, recent research suggests [39] that lipid metabolism mediated by phospholipase PLA2G2E in peri-infarct neurons might trigger endogenous neuroreparative pathways, offering a new perspective on the function of lipids during the recovery phase after stroke. Future studies are needed to explore the specific molecular mechanisms and clinical translation potential.

Sleep disorders and stroke frequently coexist [40], and post-stroke patients with poor sleep quality often face more severe rehabilitation challenges. Sleep disorders themselves constitute an important risk factor and a pre-existing pathological state for cerebrovascular disease [41]. The underlying mechanism may lie in the fact that intermittent hypoxia and sleep fragmentation triggered by sleep disturbances can impair crucial post-stroke neuroplasticity processes and exacerbate neuroinflammation, thereby creating conditions conducive to the development of spasticity. Consequently, the predictive effect of sleep disorders on PSS observed in our study likely reflects the cumulative damage to the nervous system that existed prior to the stroke onset. This pre-existing damage becomes more pronounced after the stroke event and influences the recovery trajectory. Based on this, we hypothesise that incorporating the assessment and management of sleep quality into the routine clinical pathway during post-stroke rehabilitation may hold critical significance for preventing PSS and promoting overall neurological functional recovery.

Spastic muscles are characterised by weakness [11]. Although PSS manifests as increased muscle tone, this increase represents an abnormal, uncontrolled state of tension and does not reflect a genuine enhancement of muscle strength. Early leg weakness has been found to be a major predictor of lower limb spasticity 12 months after stroke, according to Martin et al. [42]. Our multivariate analysis revealed that, compared to patients with moderate to severe muscle strength deficits (MMT score 0–3) at admission, good to normal muscle strength (MMT score 4–5) was a significant protective factor against the development of PSS, typically indicating a more favourable prognosis and a lower risk of spasticity. Muscle strength and functional health can be reliably evaluated using the MMT score at admission [43]. A lower score correlates with poorer prognosis and a higher risk of spasticity. Therefore, assessing the MMT score is crucial.

However, our research has a few shortcomings. First, it is inherently a retrospective analysis, and the effects of missing data and selection bias are unavoidable. Second, the model was developed and validated within a single-center cohort with a relatively limited sample size, which could affect the generalizability of our findings. Future research should prioritize external validation in multi-center, prospective cohorts with expanded sample sizes. Third, the definition of PSS was based on the MAS score, and the measurements were not taken at a unified fixed time point after stroke. Although our sensitivity analysis has demonstrated the robustness of the model under stricter thresholds (e.g. MAS ≥1+), potential variations in the timing of measurement and the setting of cutoff values may still pose a risk to the consistency of case classification. Additionally, although the model demonstrated excellent calibration in the training set, a calibration bias was observed in the validation set, manifesting as a slight systematic overestimation of risk. This phenomenon could be attributed to the distributional differences in demographic or clinical baseline characteristics between the validation and training cohorts, or potentially the relatively limited sample size of the validation set itself. Despite this calibration bias, the model maintained a high negative predictive value (NPV = 95.5%), underscoring its significant potential for clinical screening scenarios aimed at ruling out low-risk patients and avoiding missed diagnoses. To enhance the model’s applicability in broader populations, future work will focus on external validation in larger, multi-center prospective cohorts and consider employing post-processing calibration techniques such as Platt scaling or isotonic regression to further refine model performance. Finally, several factors (such as NHISS scores and location of occurrence) are considered to have theoretical connections to PSS and exhibited some trends of difference in descriptive statistics, but they did not reach statistical significance in multivariable analysis. This suggests that a larger sample size might be necessary to adequately power the detection of their effects. Furthermore, our research may not have explored all possible causes of PSS, and the study did not include prospective data, meaning that other significant indicators could also be potential predictors of PSS.

## 5. Conclusion

In summary, our study developed a practical predictive nomogram that identifies seven variables associated with PSS, including CRP, ALB, CK, FBG, hyperlipidemia, sleep disorders, and MMT score, to assess the risk of PSS. The principal benefit of this model resides in its ability to accurately predict PSS based on routine clinical data, making it both cost-effective and user-friendly. It is crucial to emphasise that although the nomogram developed in this study demonstrated good performance in internal validation, its clinical applicability still requires external validation through future large-scale, multi-centre, prospective studies. Prior to obtaining robust external validation evidence, this model should not be used directly as a decision-making guide for clinical treatment.

## Supplementary Material

Supplementary Material 4.docx

Supplementary Material 5.docx

Clean copy - Supplementary_Material_3 - IANN-2025-3613.R3.docx

Supplementary Material 1.docx

Supplementary Material 2.docx

## Data Availability

The underlying data for this research will be accessible upon reasonable requests to the corresponding author.

## References

[1] Global, regional, and national burden of stroke and its risk factors, 1990-2021: a systematic analysis for the global burden of disease study 2021. Lancet Neurol. 2024;23(10):973–1003. doi: 10.1016/s1474-4422(24)00369-7. 39304265 PMC12254192

[2] Feigin VL, Brainin M, Norrving B, et al. World Stroke Organization: global Stroke Fact Sheet 2025. Int J Stroke. 2025;20(2):132–144. doi: 10.1177/17474930241308142. 39635884 PMC11786524

[3] Li S. Spasticity, motor recovery, and neural plasticity after stroke. Front Neurol. 2017;8:120. doi: 10.3389/fneur.2017.00120. 28421032 PMC5377239

[4] Li S. Ankle and foot spasticity patterns in chronic stroke survivors with abnormal gait. Toxins (Basel). 2020;12(10):646. doi: 10.3390/toxins12100646. 33036356 PMC7600702

[5] Wissel J, Manack A, Brainin M. Toward an epidemiology of poststroke spasticity. Neurology. 2013;80(3 Suppl 2):S13–S19. doi: 10.1212/WNL.0b013e3182762448. 23319481

[6] Schinwelski MJ, Sitek EJ, Wąż P, et al. Prevalence and predictors of post-stroke spasticity and its impact on daily living and quality of life. Neurol Neurochir Pol. 2019;53(6):449–457. doi: 10.5603/PJNNS.a2019.0067. 31845749

[7] Sommerfeld DK, Eek EU, Svensson AK, et al. Spasticity after stroke: its occurrence and association with motor impairments and activity limitations. Stroke. 2004;35(1):134–139. doi: 10.1161/01.Str.0000105386.05173.5e. 14684785

[8] Biering-Soerensen B, Stevenson V, Bensmail D, et al. European expert consensus on improving patient selection for the management of disabling spasticity with intrathecal baclofen and/or botulinum toxin type A. J Rehabil Med. 2022;54:jrm00241. doi: 10.2340/16501977-2877. 34608495 PMC8862646

[9] Tamayo FM, Rosales R, Wissel J, et al. Botulinum toxin in pain-related post-stroke limb spasticity: a meta-analysis of early and late injections. Toxins (Basel). 2025;17(5):258. doi: 10.3390/toxins17050258. 40423340 PMC12116153

[10] Zorowitz RD, Gillard PJ, Brainin M. Poststroke spasticity: sequelae and burden on stroke survivors and caregivers. Neurology. 2013;80(3 Suppl 2):S45–S52. doi: 10.1212/WNL.0b013e3182764c86. 23319485

[11] Bavikatte G, Subramanian G, Ashford S, et al. Early identification, intervention and management of post-stroke spasticity: expert consensus recommendations. J Cent Nerv Syst Dis. 2021;13:11795735211036576. doi: 10.1177/11795735211036576. 34566442 PMC8461119

[12] Lindsay C, Ispoglou S, Helliwell B, et al. Can the early use of botulinum toxin in post stroke spasticity reduce contracture development? A randomised controlled trial. Clin Rehabil. 2021;35(3):399–409. doi: 10.1177/0269215520963855. 33040610 PMC7944432

[13] Zhu C, Li L, Qiu L, et al. Risk factors for post-stroke spasticity: a retrospective study. Front Neurol. 2024;15:1478206. doi: 10.3389/fneur.2024.1478206. 39758779 PMC11697595

[14] Burke D, Wissel J, Donnan GA. Pathophysiology of spasticity in stroke. Neurology. 2013;80(3 Suppl 2):S20–S26. doi: 10.1212/WNL.0b013e31827624a7. 23319482

[15] Li S, Francisco GE. New insights into the pathophysiology of post-stroke spasticity. Front Hum Neurosci. 2015;9:192. doi: 10.3389/fnhum.2015.00192. 25914638 PMC4392691

[16] Glaess-Leistner S, Ri SJ, Audebert HJ, et al. Early clinical predictors of post stroke spasticity. Top Stroke Rehabil. 2021;28(7):508–518. doi: 10.1080/10749357.2020.1843845. 33156735

[17] Liao LY, Xu PD, Fang XQ, et al. Prevalence and clinical predictors of spasticity after intracerebral hemorrhage. Brain Behav. 2023;13(3):e2906. doi: 10.1002/brb3.2906. 36750443 PMC10013944

[18] Chinese Medical Association Neurology Branch, Chinese Medical Association Neurology Branch Cerebrovascular Disease Group. Diagnostic criteria for various major cerebrovascular diseases in China 2019. Chinese J Neurol. 2019;52(9),710–715. doi: 10.3760/cma.j.issn.1006-7876.2019.09.003.

[19] Dunstan DA, Scott N. Clarification of the cut-off score for Zung’s self-rating depression scale. BMC Psychiatry,. 2019;19(1):177. doi: 10.1186/s12888-019-2161-0. 31185948 PMC6558728

[20] Roman NA, Miclaus RS, Nicolau C, et al. Customized manual muscle testing for post-stroke upper extremity assessment. Brain Sci. 2022;12(4):457. doi: 10.3390/brainsci12040457. 35447988 PMC9029412

[21] Kondrup J, Rasmussen HH, Hamberg O, et al. Nutritional risk screening (NRS 2002): a new method based on an analysis of controlled clinical trials. Clin Nutr. 2003;22(3):321–336. doi: 10.1016/s0261-5614(02)00214-5. 12765673

[22] Wagstaff AJ, Bryson HM. Tizanidine. A review of its pharmacology, clinical efficacy and tolerability in the management of spasticity associated with cerebral and spinal disorders. Drugs. 1997;53(3):435–452. doi: 10.2165/00003495-199753030-00007. 9074844

[23] Jia S, Liu Y, Shen L, et al. Botulinum toxin type a for upper limb spasticity in poststroke patients: a meta-analysis of randomized controlled trials. J Stroke Cerebrovasc Dis. 2020;29(6):104682. doi: 10.1016/j.jstrokecerebrovasdis.2020.104682. 32305277

[24] Afzal B, Noor R. Characterising clinical patterns of physical therapy activities for post-stroke spasticity in stroke rehabilitation: looking into the “Black Box. J Pak Med Assoc. 2024;74(4):781–784. doi: 10.47391/jpma.9040. 38751278

[25] Hurth H, Morgalla M, Heinzel J, et al. [Surgical procedures for treatment of spasticity]. Nervenarzt. 2023;94(12):1116–1122. (Chirurgische Verfahren zur Therapie von Spastik.) doi: 10.1007/s00115-023-01568-3. 37955654

[26] Stevenson VL. Rehabilitation in practice: spasticity management. Clin Rehabil. 2010;24(4):293–304. doi: 10.1177/0269215509353254. 20360150

[27] Liu H, Li J, Guo J, et al. A prediction nomogram for neonatal acute respiratory distress syndrome in late-preterm infants and full-term infants: a retrospective study. EClinicalMedicine. 2022;50:101523. doi: 10.1016/j.eclinm.2022.101523. 35784441 PMC9241127

[28] VanGilder RL, Davidov DM, Stinehart KR, et al. C-reactive protein and long-term ischemic stroke prognosis. J Clin Neurosci. 2014;21(4):547–553. doi: 10.1016/j.jocn.2013.06.015. 24211144 PMC4394376

[29] Welsh P, Barber M, Langhorne P, et al. Associations of inflammatory and haemostatic biomarkers with poor outcome in acute ischaemic stroke. Cerebrovasc Dis. 2009;27(3):247–253. doi: 10.1159/000196823. 19176958

[30] den Hertog HM, van Rossum JA, van der Worp HB, et al. C-reactive protein in the very early phase of acute ischemic stroke: association with poor outcome and death. J Neurol. 2009;256(12):2003–2008. doi: 10.1007/s00415-009-5228-x. 19609738 PMC2780652

[31] Yining G. Effect of warm acupuncture supplemented with traditional Chinese medicine decoction on neurotransmitters and apoptosis-related indexes in elderly patients with limb spasm after stroke. Chinese J Gerontol. 2020;40(16):3383–3387.

[32] Zhu Y, Xue G, Xu S, et al. U-shaped relationship of serum albumin and neurological functional outcomes after acute ischemic stroke: a prospective cohort study. Neurol Ther. 2025;14(3):949–964. doi: 10.1007/s40120-025-00729-7. 40237930 PMC12089567

[33] Norris JW, Hachinski VC, Myers MG, et al. Serum cardiac enzymes in stroke. Stroke. 1979;10(5):548–553. doi: 10.1161/01.str.10.5.548. 505497

[34] Li S, Wang A, Zhang Y, et al. Creatine kinase is associated with recurrent stroke and functional outcomes of ischemic stroke or transient ischemic attack. J Am Heart Assoc. 2022;11(6):e022279. doi: 10.1161/jaha.121.022279. 35243903 PMC9075278

[35] Meng X. Progress in rehabilitation treatment of upper limb muscle spasms in hemiplegic patients after stroke. Int J Clin Res. 2025;9(5):4–6.

[36] Rahman MH, Jha MK, Suk K. Evolving insights into the pathophysiology of diabetic neuropathy: implications of malfunctioning glia and discovery of novel therapeutic targets. Curr Pharm Des. 2016;22(6):738–757. doi: 10.2174/1381612822666151204001234. 26635266

[37] Matsumoto H, U Y. Clinical signs, neurophysiological evaluation, and medication of spasticity–review. Brain Nerve. 2008;60(12):1409–1414. 19110751

[38] Brechtel L, Poupore N, Monroe M, et al. Role of dyslipidemia in ischemic stroke patients treated in the telestroke network. Adv Med Sci. 2021;66(2):254–261. doi: 10.1016/j.advms.2021.04.003. 33940526

[39] Nakamura A, Sakai S, Taketomi Y, et al. PLA2G2E-mediated lipid metabolism triggers brain-autonomous neural repair after ischemic stroke. Neuron. 2023;111(19):2995–3010.e9. e2999. doi: 10.1016/j.neuron.2023.06.024. 37490917

[40] Mims KN, Kirsch D. Sleep and Stroke. Sleep Med Clin. 2016;11(1):39–51. doi: 10.1016/j.jsmc.2015.10.009. 26972032

[41] Mc Carthy CE, Yusuf S, Judge C, et al. Sleep patterns and the risk of acute stroke: results from the INTERSTROKE international case-control study. Neurology. 2023;100(21):e2191–e2203. doi: 10.1212/wnl.0000000000207249. 37019662 PMC10238154

[42] Martin A, Abogunrin S, Kurth H, et al. Epidemiological, humanistic, and economic burden of illness of lower limb spasticity in adults: a systematic review. Neuropsychiatr Dis Treat. 2014;10:111–122. doi: 10.2147/ndt.S53913. 24482572 PMC3905098

[43] Spirina MA, Vlasova TI, Sitdikova AV, et al. [Neurophysiological substantiation and validity assessment of manual muscle testing in clinical practice. (A literature review)]. Vopr Kurortol Fizioter Lech Fiz Kult. 2024;102(4):70–77.(klinicheskoi praktike. (Obzor literatury).) doi: 10.17116/kurort202410104170. 39248589

