# Supplementary material for: Construction and verification of a nomogram model for predicting the risk of post-stroke spasticity: a retrospective study
Source: Ann Med. 2025 Dec 23;58(1):2604857. doi: 10.1080/07853890.2025.2604857 (PMC12777886; doi:10.1080/07853890.2025.2604857)

**Supplementary Material 4: Comparison of ROC values before and after the definition of data adjustment in sensitivity analysis**


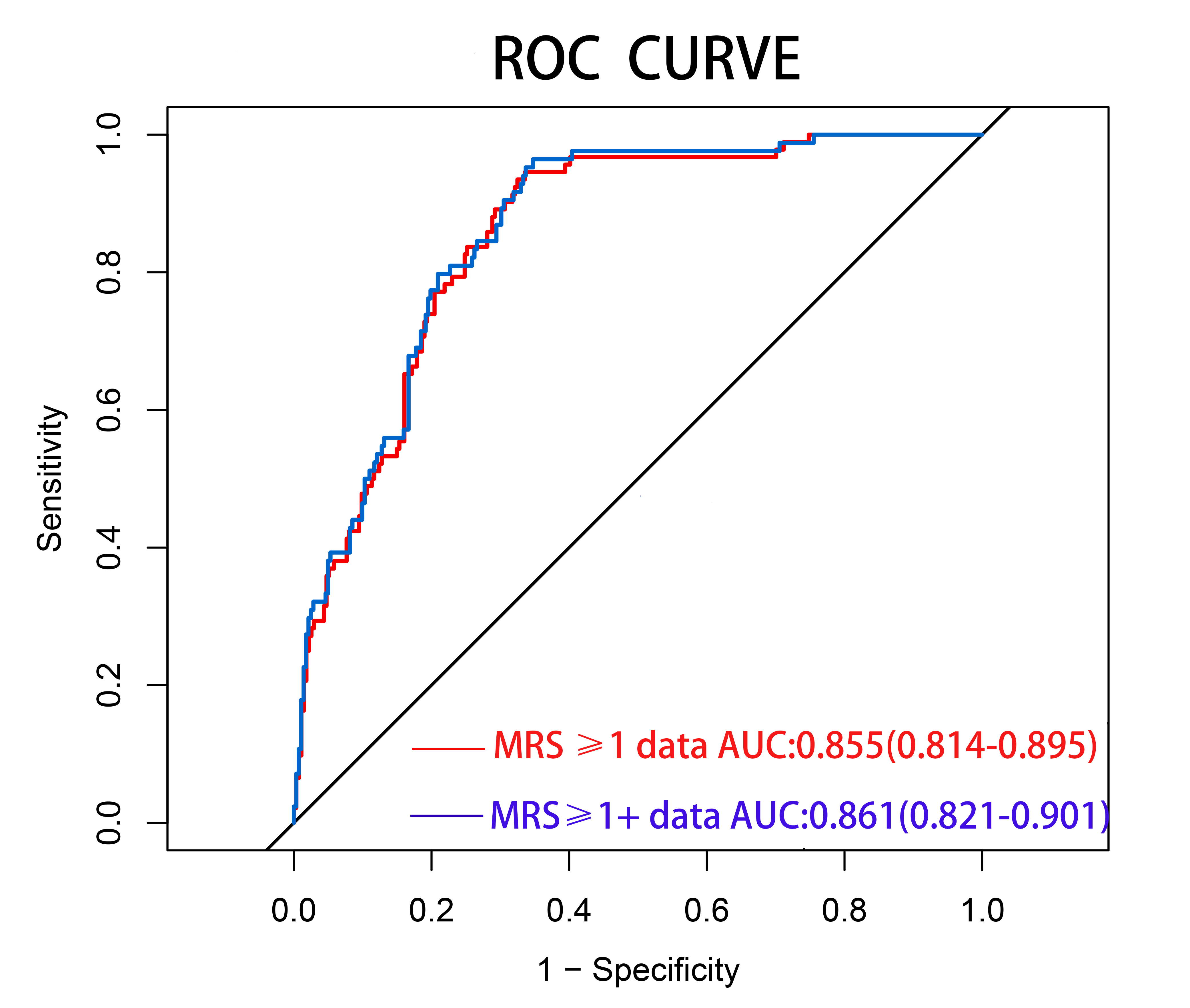

Supplement: Supplementary Material 4.docx [file IANN_A_2604857_SM7777.docx]
