# Supplementary material for: Construction and verification of a nomogram model for predicting the risk of post-stroke spasticity: a retrospective study
Source: Ann Med. 2025 Dec 23;58(1):2604857. doi: 10.1080/07853890.2025.2604857 (PMC12777886; doi:10.1080/07853890.2025.2604857)
Supplement: Supplementary Material 5.docx [file IANN_A_2604857_SM7776.docx]

**Supplementary Material 5: Results of cross-validation and bootstrap analysis**

| **measures** | **fold cross-validation(CI 95%)** | **bootstrap optimism-corrected(CI 95%)** |
| --- | --- | --- |
| auroc | 0.840(0.768-0.912) | 0.823(0.775-0.871) |
| sensitivity | 0.915(0.858-0.971) | 0.882(0.840-0.921) |
| specificity | 0.506(0.469-0.646) | 0.525(0.416-0.646) |
| accuracy | 0.789(0.712-0.865) | 0.785(0.743-0.829) |
| precision | 0.816(0.732-0.899) | 0.834(0.792-0.879) |
| recall | 0.915(0.858-0.971) | 0.882(0.840-0.921) |

OR = odds ratio; CI = confidence interval.
