# Supplementary material for: Construction and verification of a nomogram model for predicting the risk of post-stroke spasticity: a retrospective study
Source: Ann Med. 2025 Dec 23;58(1):2604857. doi: 10.1080/07853890.2025.2604857 (PMC12777886; doi:10.1080/07853890.2025.2604857)
Supplement: Clean copy - Supplementary_Material_3 - IANN-2025-3613.R3.docx [file IANN_A_2604857_SM7775.docx]

# Supplementary Material 3: Complete Logistic Regression Model Equation for Post-Stroke Spasticity Prediction

## ABSTRACT

This supplementary material provides the complete mathematical specification of the logistic regression model developed to predict post-stroke spasticity (PSS) within 3 months following acute stroke. All regression coefficients, including the intercept term, are reported with precision sufficient for independent model validation and implementation.

## 1. MODEL SPECIFICATION

### 1.1 Mathematical Formula

The probability of developing PSS is calculated using the following logistic regression equation:

*logit(P) = β₀ + β₁×CRP + β₂×Albumin + β₃×CK + β₄×FBG + β₅×Hyperlipidemia + β₆×Sleep_disorder + β₇×MMT*

The predicted probability is derived through the inverse logit transformation:

*P(PSS) = 1 / [1 + exp(-logit(P))]*

where *P(PSS)* represents the probability of developing post-stroke spasticity within 3 months.

### 1.2 Model Development

The model was developed using least absolute shrinkage and selection operator (LASSO) regression for variable selection, followed by multivariable logistic regression. The final model included seven predictors with no significant multicollinearity (all VIF < 5).

## 2. REGRESSION COEFFICIENTS

| **Variable** | **Coefficient (β)** | **Standard Error** | **Wald Z** | **P-value** | **Odds Ratio** | **95% CI** |
| --- | --- | --- | --- | --- | --- | --- |
| **Intercept (β₀)** | -9.5300 | 2.0737 | -4.60 | <0.0001 | - | - |
| CRP (mg/L) | 0.1478 | 0.0410 | 3.61 | 0.0003 | 1.159 | 1.070-1.256 |
| Albumin (g/L) | 0.1262 | 0.0461 | 2.74 | 0.0061 | 1.135 | 1.037-1.242 |
| Creatine Kinase (U/L) | 0.0093 | 0.0027 | 3.43 | 0.0006 | 1.009 | 1.004-1.015 |
| Fasting Blood Glucose (mmol/L) | 0.3151 | 0.1039 | 3.03 | 0.0024 | 1.370 | 1.118-1.680 |
| Hyperlipidemia (Yes vs No) | -1.2751 | 0.5447 | -2.34 | 0.0192 | 0.279 | 0.096-0.813 |
| Sleep Disorder (Yes vs No) | 1.5727 | 0.3679 | 4.27 | <0.0001 | 4.819 | 2.343-9.912 |
| MMT Score (Grade 0-3 vs 4-5) | -1.2603 | 0.3978 | -3.17 | 0.0015 | 0.284 | 0.130-0.618 |

**Model Statistics:** Likelihood Ratio χ² = 85.95 (df = 7, P < 0.0001); C-statistic = 0.844; R² = 0.413; Brier Score = 0.135

## 3. VARIABLE DEFINITIONS AND CODING

### 3.1 Continuous Variables

All continuous variables are entered as original measured values without standardization or transformation:

- **CRP (C-Reactive Protein):** Measured in mg/L using standard immunoturbidimetric assay
- **Albumin:** Measured in g/L using bromocresol green method
- **CK (Creatine Kinase):** Measured in U/L using enzymatic rate method
- **FBG (Fasting Blood Glucose):** Measured in mmol/L using glucose oxidase method

### 3.2 Categorical Variables

Binary coding scheme for categorical predictors:

- **Hyperlipidemia:** 0 = Absent, 1 = Present (diagnosed according to medical history or laboratory criteria)
- **Sleep Disorder:** 0 = Absent (PSQI ≤7), 1 = Present (PSQI >7 at admission)
- **MMT Score:** 0 = Grade 0-3 (moderate to severe muscle weakness), 1 = Grade 4-5 (good to normal muscle strength)

## 4. WORKED EXAMPLE

### 4.1 Case Description

A representative patient case from Section 3.3 of the main manuscript:

| **Variable** | **Value** |
| --- | --- |
| CRP | 9 mg/L |
| Albumin | 46 g/L |
| CK | 340 U/L |
| FBG | 6.0 mmol/L |
| Hyperlipidemia | Yes (coded as 1) |
| Sleep Disorder | Yes (coded as 1) |
| MMT Score | Grade 0-3 (coded as 0) |

### 4.2 Step-by-Step Calculation

**Step 1: Calculate logit(P)**

*logit(P) = -9.5300 + 0.1478×9 + 0.1262×46 + 0.0093×340 + 0.3151×6.0 + (-1.2751)×1 + 1.5727×1 + (-1.2603)×0*

Computing each component:

- Intercept: -9.5300
- CRP contribution: 0.1478 × 9 = 1.3302
- Albumin contribution: 0.1262 × 46 = 5.8052
- CK contribution: 0.0093 × 340 = 3.1620
- FBG contribution: 0.3151 × 6.0 = 1.8906
- Hyperlipidemia contribution: -1.2751 × 1 = -1.2751
- Sleep disorder contribution: 1.5727 × 1 = 1.5727
- MMT contribution: -1.2603 × 0 = 0.0000

*logit(P) = -9.5300 + 1.3302 + 5.8052 + 3.1620 + 1.8906 - 1.2751 + 1.5727 + 0.0000 = 2.9556*

**Step 2: Calculate Predicted Probability**

*P(PSS) = 1 / [1 + exp(-2.9556)] = 1 / [1 + 0.0519] = 1 / 1.0519 = 0.9507*

**Result:** The predicted probability of developing PSS for this patient is **95.1%**.

**Note on Numerical Precision:**

The predicted probability calculated using the exact regression coefficients is 95.1%. The value of 92.5% reported in Section 3.3 of the main manuscript was derived from visual estimation using the nomogram graphical representation. This 2.6 percentage point discrepancy arises from: (1) rounding of coefficients in the nomogram display for visual clarity, (2) inherent measurement imprecision when reading values from graphical scales, and (3) slight variations in numerical precision between plotting software and direct calculation.

Both values consistently indicate high risk (>90% probability) and lead to the same clinical interpretation. This magnitude of discrepancy is within the expected and acceptable range for nomogram-based predictions. For applications requiring maximum precision, we recommend using either the exact formula provided in this supplementary material or the web-based calculator (https://newpredict.shinyapps.io/DynNomapp2/).

## 5. MODEL PERFORMANCE METRICS

| **Metric** | **Training Set (n=257)** | **Validation Set (n=109)** |
| --- | --- | --- |
| AUC (95% CI) | 0.844 (0.793-0.896) | 0.842 (0.765-0.920) |
| Optimal Cut-off | 0.249 | 0.189 |
| Sensitivity | 81.2% | 87.0% |
| Specificity | 76.1% | 74.4% |
| Positive Predictive Value | 55.4% | 51.6% |
| Negative Predictive Value | 91.7% | 95.5% |
| Hosmer-Lemeshow Test | P = 0.944 | P = 0.157 |
| Brier Score | 0.135 | 0.133 |

## 6. CLINICAL APPLICATION GUIDELINES

- **High Risk (P ≥ 0.65):** Recommend aggressive preventive interventions, including early consideration of botulinum toxin injection and intensive rehabilitation protocols
- **Moderate Risk (0.25 ≤ P < 0.65):** Close monitoring with individualized management based on clinical progression and patient-specific factors
- **Low Risk (P < 0.25):** Routine rehabilitation care with periodic reassessment at regular intervals

### 7. MODEL CHARACTERISTICS

- The model uses original (non-standardized) variable values
- No data transformation or standardization is required for application
- Missing data should not be imputed arbitrarily; patients with missing key predictors should be excluded from prediction
- The model predicts PSS risk within 3 months post-stroke; applicability beyond this timeframe requires further validation

## 8. STUDY LIMITATIONS

- The model was developed using single-center retrospective data from a Chinese population
- External validation in independent prospective cohorts from diverse geographic and ethnic populations is required
- The model does not account for post-admission interventions or changes in patient status
- Clinical decisions should integrate model predictions with physician clinical judgment and patient-specific circumstances

## 9. REPORTING STANDARDS

This model was developed and reported following the Transparent Reporting of a multivariable prediction model for Individual Prognosis Or Diagnosis (TRIPOD) guidelines for prediction model development and validation.

## 10. CONTACT INFORMATION

**Corresponding Author:** Shu Xiong Luo, MD
**Email:** 289611595@qq.com
**Institution:** Department of Tuina, Dongguan Hospital of Traditional Chinese Medicine, Guangzhou University of Chinese Medicine, Dongguan, China

For inquiries regarding:

- Model implementation or technical details
- Access to de-identified training data for external validation
- Clarification of variable measurement protocols

Please contact the corresponding author.
