# Supplementary material for: Construction and verification of a nomogram model for predicting the risk of post-stroke spasticity: a retrospective study
Source: Ann Med. 2025 Dec 23;58(1):2604857. doi: 10.1080/07853890.2025.2604857 (PMC12777886; doi:10.1080/07853890.2025.2604857)
Supplement: Supplementary Material 1.docx [file IANN_A_2604857_SM7774.docx]

### **Supplementary Material 1:**

### **Handling of Missing Data**

1. **Assessment of Missing Data**

Prior to analysis, we assessed the pattern and extent of missingness in the candidate predictor variables among the initial study cohort. Only three variables were found to have missing values: Glycated hemoglobin (HbA1c) was missing in 13 cases (3.6%), Lactate dehydrogenase in 25 cases (6.8%), and Magnesium in 9 cases (2.5%). The overall missing data rate for each variable was below 10%, which is considered acceptable for employing multiple imputation techniques rather than complete-case analysis, thereby preserving statistical power and reducing potential bias ^[1,2]^.

1. **Imputation Strategy**

To handle the missing data, we employed Multiple Imputation (MI) using the SPSS software (version 27.0). The imputation process was based on the Fully Conditional Specification (FCS) algorithm, also known as Multiple Imputation by Chained Equations (MICE). This method iteratively imputes missing values for each variable conditional on the other variables in the dataset, which is particularly suitable for mixed data types (continuous and categorical). We generated 5 imputed datasets over 5 iterations. The results from these datasets were then pooled for the final analysis according to Rubin's rules, which appropriately reflects the uncertainty introduced by the missing data .

1. **Sensitivity Analysis for Robustness**

To rigorously evaluate the impact of the missing data and the imputation method on our primary findings, we conducted a sensitivity analysis by constructing a "complete-case dataset." This dataset was created by excluding 13 cases missing HbA1c, 25 cases missing lactate dehydrogenase, and 9 cases missing magnesium. The primary prediction model was then rebuilt using this dataset.

1. **Results of Sensitivity Analysis**

The performance of the model derived from the imputed data was compared with that from the complete-case dataset. As illustrated in the Supplementary Figure 1 below, the predictive performance, measured by the Area Under the Receiver Operating Characteristic Curve (AUC), was nearly identical between the two approaches:

- Complete-Case Model AUC: 0.855 (95% CI: 0.814 - 0.895)
- Imputed Data Model AUC: 0.857 (95% CI: 0.816 - 0.898)

**Supplementary Figure 1. Comparison of ROC values before and after data imputation in sensitivity analysis**


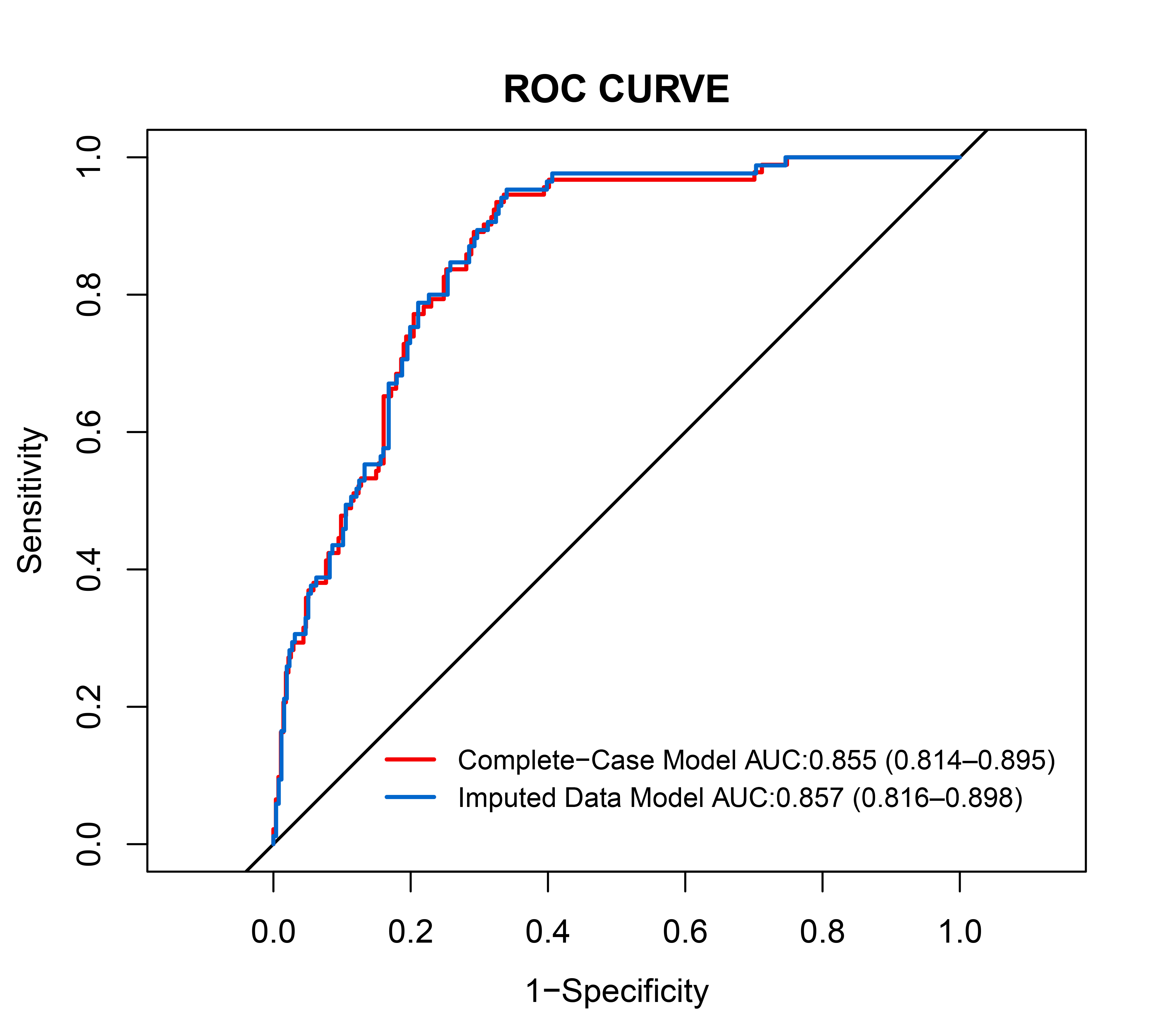


The close agreement in AUC values indicates that the multiple imputation method had a negligible impact on the model's predictive performance. This high level of consistency confirms that the core conclusions of our study are robust to the method used for handling missing data.

**References:**

[1] Sterne, J. A., White, I. R., Carlin, J. B., Spratt, M., Royston, P., Kenward, M. G., Wood, A. M., & Carpenter, J. R. (2009). Multiple imputation for missing data in epidemiological and clinical research: potential and pitfalls. *Bmj*, *338*, b2393. <https://doi.org/10.1136/bmj.b2393>

[2] Jakobsen, J. C., Gluud, C., Wetterslev, J., & Winkel, P. (2017). When and how should multiple imputation be used for handling missing data in randomised clinical trials - a practical guide with flowcharts. *BMC Med Res Methodol*, *17*(1), 162. <https://doi.org/10.1186/s12874-017-0442-1>
