# Supplementary material for: Construction and verification of a nomogram model for predicting the risk of post-stroke spasticity: a retrospective study
Source: Ann Med. 2025 Dec 23;58(1):2604857. doi: 10.1080/07853890.2025.2604857 (PMC12777886; doi:10.1080/07853890.2025.2604857)
Supplement: Supplementary Material 2.docx [file IANN_A_2604857_SM7773.docx]

**Supplementary Material 2: Multicollinearity test**

**Supplementary Table 1. Results of multicollinearity test**

| Variables | VIF | 1/VIF |
| --- | --- | --- |
| Age | 1.170 | 0.855 |
| Gender | 1.316 | 0.760 |
| Hypertension | 1.185 | 0.844 |
| Diabetes | 1.155 | 0.866 |
| Hyperlipidemias | 1.163 | 0.860 |
| Smoking | 1.139 | 0.878 |
| Types of stroke | 1.254 | 0.797 |
| Location of occlusion | 1.154 | 0.867 |
| Hemorrhagic or Ischemic | 1.154 | 0.867 |
| Side of infarction or hemorrhage | 1.159 | 0.863 |
| Large area of infarction | 1.244 | 0.804 |
| MMT | 1.173 | 0.853 |
| Sleep disorders | 1.159 | 0.863 |
| Depression | 1.151 | 0.869 |
| ADL | 1.156 | 0.865 |
| NRS | 1.159 | 0.863 |
| NIHSS | 1.209 | 0.826 |
| WBC | 1.722 | 0.581 |
| CRP | 1.656 | 0.604 |
| Albumin | 1.347 | 0.742 |
| HbA1c | 1.311 | 0.763 |
| FBG | 1.314 | 0.761 |
| CK | 1.223 | 0.818 |
| LDH | 1.205 | 0.830 |
| K | 1.213 | 0.824 |
| Mg | 1.170 | 0.855 |
| Ca | 1.167 | 0.857 |

**Abbreviations:**  VIF,Variance Inflation Factor.​

**Supplementary Table 2. Multicollinearity test for predictor variables​**

| Variables | VIF | 1/VIF |
| --- | --- | --- |
| Hyperlipidemias | 1.064 | 0.940 |
| MMT | 1.052 | 0.951 |
| Sleep disorders | 1.051 | 0.951 |
| CRP | 1.050 | 0.952 |
| Albumin | 1.084 | 0.923 |
| FBG | 1.025 | 0.976 |
| CK | 1.066 | 0.938 |
